# Predictive accuracy of biomarkers for survival among cardiac arrest patients with hypothermia: a prospective observational cohort study in Japan

**DOI:** 10.1186/s13049-020-00765-2

**Published:** 2020-08-05

**Authors:** Yohei Okada, Takeyuki Kiguchi, Taro Irisawa, Kazuhisa Yoshiya, Tomoki Yamada, Koichi Hayakawa, Kazuo Noguchi, Tetsuro Nishimura, Takuya Ishibe, Yoshiki Yagi, Masafumi Kishimoto, Hiroshi Shintani, Yasuyuki Hayashi, Taku Sogabe, Takaya Morooka, Haruko Sakamoto, Keitaro Suzuki, Fumiko Nakamura, Norihiro Nishioka, Tasuku Matsuyama, Satoshi Matsui, Takeshi Shimazu, Kaoru Koike, Takashi Kawamura, Tetsuhisa Kitamura, Taku Iwami

**Affiliations:** 1 Department of Preventive Services, School of Public Health, Kyoto University, Kyoto, 606-8501 Japan; 2 Department of Primary care and Emergency Medicine, Graduate School of Medicine, Kyoto University, Kyoto, Japan; 3 Kyoto University Health Services, Kyoto, Japan; 4 Critical Care and Trauma Center, Osaka General Medical Center, Osaka, Japan; 5 Department of Traumatology and Acute Critical Medicine, Osaka University Graduate School of Medicine, Suita, Japan; 6 Emergency and Critical Care Medical Center, Osaka Police Hospital, Osaka, Japan; 7 Department of Emergency and Critical Care Medicine, Kansai Medical University, Takii Hospital, Moriguchi, Japan; 8 Department of Emergency Medicine, Tane General Hospital, Osaka, Japan; 9 Department of Critical Care Medicine, Osaka City University, Osaka, Japan; 10 Department of Emergency and Critical Care Medicine, Kinki University School of Medicine, Osaka-Sayama, Japan; 11 Osaka Mishima Emergency Critical Care Center, Takatsuki, Japan; 12 Osaka Prefectural Nakakawachi Medical Center of Acute Medicine, Higashi-Osaka, Japan; 13 Senshu Trauma and Critical Care Center, Osaka, Japan; 14 Senri Critical Care Medical Center, Saiseikai Senri Hospital, Suita, Japan; 15 Traumatology and Critical Care Medical Center, National Hospital Organization Osaka National Hospital, Osaka, Japan; 16 Emergency and Critical Care Medical Center, Osaka City General Hospital, Osaka, Japan; 17 Department of Pediatrics, Osaka Red Cross Hospital, Osaka, Japan; 18 Emergency and Critical Care Medical Center, Kishiwada Tokushukai Hospital, Osaka, Japan; 19 Department of Emergency and Critical Care Medicine, Kansai Medical University, Hirakata, Osaka, Japan; 20 Department of Emergency Medicine, Kyoto Prefectural University of Medicine, Kyoto, Japan; 21 Division of Environmental Medicine and Population Sciences, Department of Social and Environmental Medicine, Graduate School of Medicine, Osaka University, Osaka, Japan

**Keywords:** Out-of-hospital cardiac arrest, Hypothermia, Diagnosis, Prediction, Prognosis

## Abstract

**Background:**

There is limited information on the predictive accuracy of commonly used predictors, such as lactate, pH or serum potassium for the survival among out-of-hospital cardiac arrest (OHCA) patients with hypothermia. This study aimed to identify the predictive accuracy of these biomarkers for survival among OHCA patients with hypothermia.

**Methods:**

In this retrospective analysis, we analyzed the data from a multicenter, prospective nationwide registry among OHCA patients transported to emergency departments in Japan (the JAAM-OHCA Registry). We included all adult (≥18 years) OHCA patients with hypothermia (≤32.0 °C) who were registered from June 2014 to December 2017 and whose blood test results on hospital arrival were recorded. We calculated the predictive accuracy of pH, lactate, and potassium for 1-month survival.

**Results:**

Of the 34,754 patients in the JAAM-OHCA database, we included 754 patients from 66 hospitals. The 1-month survival was 5.8% (44/754). The areas under the curve of the predictors and 95% confidence interval were as follows: pH 0.829 [0.767–0.877] and lactate 0.843 [0.793–0.882]. On setting the cutoff points of 6.9 in pH and 120 mg/dL (13.3 mmol/L) in lactate, the predictors had a high sensitivity (lactate: 0.91; pH 0.91) and a low negative likelihood ratio (lactate: 0.14; pH 0.13), which are suitable to exclude survival to 1 month. Furthermore, in additional analysis that included only the patients with potassium values available, a cutoff point of 7.0 (mmol/L) for serum potassium had high sensitivity (0.96) and a low negative likelihood ratio (0.09).

**Conclusion:**

This study indicated the predictive accuracy of serum lactate, pH, and potassium for 1-month survival among adult OHCA patients with hypothermia. These biomarkers may help define a more appropriate resuscitation strategy.

## Background

Accidental hypothermia is defined as an involuntary drop of the core body temperature (BT) below 35 °C and is associated with significant morbidity and mortality [1]. Particularly, in moderate-to-severe hypothermia of less than 32 °C, the risk of arrhythmia and cardiac arrest increases [1]. For such patients who progressed to cardiac arrest, the guidelines suggest extracorporeal life support (ECLS) and aggressive internal rewarming by using veno-arterial extracorporeal membrane oxygenation (V-A ECMO) based on the classic dictum that “No one is dead until warm and dead” [2, 3]. This is because cardiac arrest patients with hypothermia have been reported to achieve good clinical outcomes even if the cardiac arrest was sustained for a long duration [2–6].

However, most of the patients in these previous reports comprised younger populations associated with outdoor activities such as skiing or climbing in winter [7–10]. In such patients, aggressive application of advanced resuscitation may be acceptable. Conversely, in a super-aging society such as Japan, most of the patients of accidental hypothermia are elderly and frail and found in indoor settings [11, 12]. For such a population, ECLS might possibly become an undesirable life-sustaining treatment in some cases; thus, the decision for implementation of ECLS should be taken more cautiously. Accurate prediction and identification of the patients who are likely to have good or bad outcomes can facilitate the selection of patients for ECLS or termination of resuscitation. Thus, an accurate predictor of the outcome is necessary among cardiac arrest patients with hypothermia.

Some biomarkers such as lactate, pH, and serum potassium are well-known, good predictors of clinical outcome, and these are suggested to be considered for implementation of ECLS or its termination according to the European Resuscitation Council guidelines for hypothermic patients [1–3, 7–10, 13]. However, little is known about the predictive accuracy of these parameters because these suggestions were based on case reports or cohort studies with a small sample size in patients of cardiac arrest with hypothermia. Therefore, it is valuable to assess the predictive accuracy of the predictors in a large cohort study comprising many out-of-hospital cardiac arrest (OHCA) patients with hypothermia. This study was conducted with an aim to identify the predictive accuracy of serum biomarkers with regard to survival among OHCA patients with hypothermia.

## Methods

The methodology of this study is reported in accordance with the Standards for Reporting of Diagnostic Accuracy Studies (STARD) 2015 guidelines [14]. This study was approved by the ethics committee of Kyoto University (R-1045). The need for informed consent was waived in view of the study design.

### Study design and settings

We undertook this study to identify the diagnostic accuracy of specific clinical parameters through a retrospective analysis of the JAAM-OHCA Registry [15], a multicenter, prospective nationwide database that includes pre-hospital information, in-hospital information, and outcome among OHCA patients transported to emergency departments in Japan. Details about this registry have been previously reported [15, 16]. The JAAM-OHCA Registry was established in 2014 by the organizing committee of the registry to improve therapeutic strategy, emergency medical systems, and patient outcome. Presently, the registry includes 87 institutions, and 66 of the included hospitals are university hospitals and/or critical care centers. These critical care centers were certified by the Ministry of Health, Labor, and Welfare in Japan, and they are equipped to provide highly specialized treatment, such as ECLS, percutaneous coronary intervention, or targeted 24-h temperature management. The other 21 hospitals were not certified as critical care centers, but provided emergency medical service to the community. A total of 34,754 OHCA patients were registered in the JAAM-OHCA Registry from June 2014 to December 2017.

Prehospital information was collected by paramedics based on the standardized Utstein format [17], and verified by the Fire and Disaster Management Agency in Japan. In-hospital information was registered by clinicians or clinical data administrators at each institution, using a standardized online form. The in-hospital information has a fundamental and supplemental variables section. Fundamental variables (e.g., basic characteristics, blood gas assessment data, and outcome) were mandatorily registered in all cases if available, while supplemental variables (e.g., blood chemistry data) were recorded if the institutions applied additional protocols and recorded them. The JAAM-OHCA registry committee combined the in-hospital and prehospital information and logically evaluated the data quality. Finally, de-identified data were provided to the researchers by the registry’s committee.

### Participants

We included all adult (≥18 years old) OHCA patients transported to emergency departments with moderate-to-severe hypothermia and registered in the database from June 2014 to December 2017. Moderate-to-severe hypothermia was defined as BT 32 °C or lower on hospital arrival, based on the Swiss grading system [18] and the related guidelines [1–3]. In general, it is challenging to differentiate whether the cardiac arrest was primarily caused by hypothermia precisely, or the cardiac arrest patient became hypothermic on hospital arrival; thus, we did not attempt to distinguish these two states. We excluded patients who received no resuscitation attempts in the hospital. This is because those patients were obviously dead, as evidenced by the existence of postpartum changes such as rigor mortis, or the patients had already documented a “do not resuscitate” order. Moreover, we excluded patients who opted out from participation in the study, and patients who were cases of obvious traumatic cardiac arrest or hanging, who had no pre-hospital data, no BT, and had no blood tests conducted. Furthermore, as explained earlier, age, BT, lactate, and pH values were fundamental variables; however, since potassium was a supplemental variable, it was only available in the data from institutions that applied additional protocols. Thus, to analyze the predictive value of potassium, we undertook additional analysis to exclude the patients who were transferred to institutions that did not apply the additional research protocol to record the serum potassium values.

### Index test

Based on reports in the previous literature [1, 2, 7–9, 13, 19–21], we selected three potential predictors: serum pH, lactate, and potassium values. These values were defined as the measurements from the initial blood test or blood biochemistry tests conducted on hospital arrival in the emergency department. Moreover, we selected age and BT as a reference, and BT was defined as the body temperature measured initially on hospital arrival.

### Target condition

The primary target condition to be predicted in this study was the 1-month survival.

### Statistical analysis

#### Patient and hospital characteristics

We described the patients and hospital characteristics as follows: sex age, season (Spring: March–June; Summer: July–August; Autumn: September–November; and Winter: December–February), and regions of Japan (Northern, Eastern, Western, and Southern). The season and area were defined by the definition of the Japan Meteorological Agency (details of the area are described in the supplementary materials) [22]. In addition, we described pre-hospital and in-hospital patient data as follows: bystander witness, bystander CPR, shockable on initial rhythm, advanced airway inserted by paramedics, cardiac rhythm on hospital arrival [return of spontaneous circulation (ROSC), shockable, pulseless electrical activity (PEA), asystole], BT on arrival, and ECMO implementation. We also included blood test results on arrival, time course (from emergency call to hospital arrival to blood test to ROSC and/or to ECMO), and the disposition (admit to intensive care unit (ICU)/ward, or death in the emergency department]. A shockable rhythm was defined as ventricular fibrillation (VF) or pulseless ventricular tachycardia (VT). ROSC was defined as the presence of a palpable pulse for more than 30 s despite circulatory support by ECMO [23]. Furthermore, we indicated the hospital’s basic information as follows: type of hospitals (whether a tertiary-care center) and the number of beds. A tertiary center was defined as university hospitals and/or critical care centers certified by the government, as explained earlier. Data were presented as median and interquartile range (IQR) for continuous variables, and as number and percentages for categorical variables; missing values are shown as “Missing” or “Unknown.”

#### Predictive accuracy

We calculated the predictive accuracy of pH, and lactate on target conditions in the study population. We also calculated the accuracy of age and BT as a reference. We showed the discriminatory ability of each predictor for 1-month survival by using the receiver-operating characteristic curve (ROC) and area under the curve (AUC) with 95% confidence interval (CI). Moreover, we set the cutoff value and created 2 × 2 tables to calculate sensitivity (Se), specificity (Sp), positive and negative likelihood ratio (LR+ and LR−, respectively), and positive and negative predictive value (PPV and NPV, respectively). In general, a pre-specified cutoff value was recommended to estimate the diagnostic accuracy [14]. The cutoff values were suggested as a serum potassium level of 8 or 10 mmol/L and pH 6.5 in the published literature [2, 3, 8, 9, 13], although these have not been specifically established. Therefore, we specified several rounded-off values in the range of interest as the cutoff points to avoid optimistic interpretation and facilitate ease of clinical use [14]. Furthermore, we suggested cutoff values in each predictor on the basis of the LR+ and LR− (LR+, > 5; LR−, < 0.2), which are commonly thought to be useful for rule-in or rule-out decision making [24]. We did not undertake a sample-size estimation, because it was the secondary usage of an already available database and a retrospective analysis. Missing data were handled by the exclusion of that specific patient to calculate the predictive accuracy. All statistical results were considered significant at a two-sided *P*-value of < 0.05. All statistical analyses were undertaken in JMP Pro® 14 software (SAS Institute Inc., Cary, NC, USA).

#### Additional analysis

As mentioned earlier, a substantial number of institutions did not apply the additional protocol to record potassium values. Thus, we conducted additional analyses to calculate the predictive accuracy of potassium and other predictors after excluding the patients who were transferred to the institutions without an additional protocol to record the potassium data.

## Results

### Study participants

**Fig. 1:**
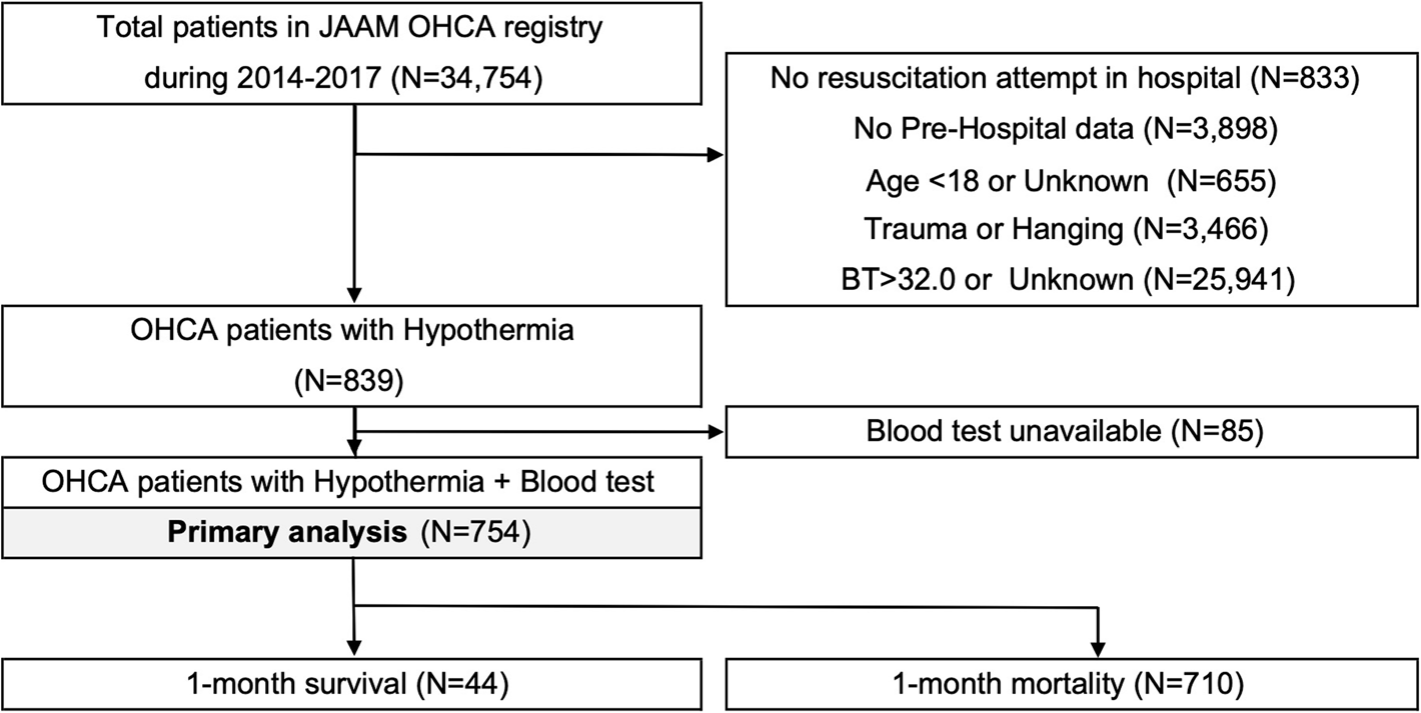
Study flowchart. OHCA, Out-of-hospital cardiac arrest; BT, Body temperature

Among the 34,754 patients in the JAAM-OHCA database, 754 patients whose blood gas assessment results were available from 66 hospitals (tertiary center 56, non-tertiary center 10 hospitals) were included in the primary analysis (Fig. 1). The characteristics of the patients and in-hospital data are described in Tables 1 and 2. Briefly stated, the median [IQR] of the age are 75 [64–84] years, and almost half of the cases happened in winter (348/754, 46.2%) and northern area (303/754, 40.2%); most of the cases occurred in seasons other than summer (670/754, 88.9%) overall in spring, autumn, and winter. The median [IQR] of BT was 30.0 °C [26.4–31.3]. The 1-month survival was 5.8% (44/754).

**Table 1:** Patient characteristics

Variables	Total (*N* = 754)
**Baseline characteristics**	
Sex (Men)	448 (59.4%)
Age (years)	75 [64–84]
16–64	191 (25.3%)
65–74	181 (24%)
≥75	382 (50.7%)
**Pre-hospital information**	
Bystander witness	208 (27.6%)
Bystander CPR	276 (36.6%)
Shockable on initial rhythm	62 (8.22%)
Advanced airway	375 (49.7%)
**In-hospital information**	
Body temperature	30 [26.4–31.3]
Measurement site	
Rectal	151 (20%)
Bladder	95 (12.6%)
Tympanic	49 (6.5%)
Other/unknown	459 (60.9%)
Cardiac rhythm on hospital arrival	
ROSC	32 (4.24%)
Shockable	57 (7.56%)
PEA	128 (17%)
Asystole	537 (71.2%)
ECMO implementation	59 (7.82%)
Before ROSC	48
ROSC after hospital arrival	157 (20.8%)
Time course (min)	
E-call to hospital arrival	34 [29–43]
E-call to blood test	41 [35–52]
E-call to ECMO	70 [51.8–88]
E-call to ROSC after arrival	51 [40–85]
Blood test on hospital arrival	
pH	6.8 [6.63–6.97]
(Missing)	50 (6.6%)
Lactate (mg/dL)	135 [90.9–180]
(Missing)	53 (7.0%)
Potassium (mmol/L)	6.6 [4.9–9.6]
(Missing)	383 (50.8%)
Outcomes	
Admission to ICU or ward	152 (20.2%)
Death in ER	602 (79.8%)
1-month survival	44 (5.8%)
1-month CPC1,2	24 (3.2%)

Continuous variables are described as median [Interquartile range (IQR)]. Categorical variables are described as number (%). Shockable: ventricular fibrillation and pulseless ventricular tachycardia, *CPR* Cardiopulmonary resuscitation, *E-call* Emergency call for ambulance, *ROSC* Return of spontaneous circulation, *PEA* Pulseless electrical activity, *ECMO* Extracorporeal membrane oxygenation, *ER* Emergency room, *CPC* Cerebral performance category [17]

**Table 2:** Hospital characteristics, geographical information, and season

Variables	Total (***N*** = 754)
**Hospital Information**	
**Hospital**	
Tertiary center (56 hospitals)	727 (96.3%)
Non-tertiary center (10 hospitals)	28 (3.7%)
Number of beds	678 [561–750]
**ECMO availability**	
Always	634 (84%)
Partial	101 (13.4%)
Unavailable	20 (2.6%)
**Geographical information and season**	
**Area**	
Northern area	303 (40.1%)
Eastern area	267 (35.4%)
Western area	185 (24.5%)
Southern area	0 (0%)
**Season**	
Spring	84 (11.1%)
Summer	175 (23.2%)
Autumn	147 (19.5%)
Winter	348 (46.2%)

Continuous variables are described as median [Interquartile range (IQR)]. Categorical variables are expressed as number (%). Shockable: ventricular fibrillation and pulseless ventricular tachycardia

*E-call* Emergency call for ambulance, *ROSC* Return of spontaneous circulation, *ECMO* Extracorporeal membrane oxygenation, *ER* Emergency room

### Predictive accuracy

**Fig. 2:**
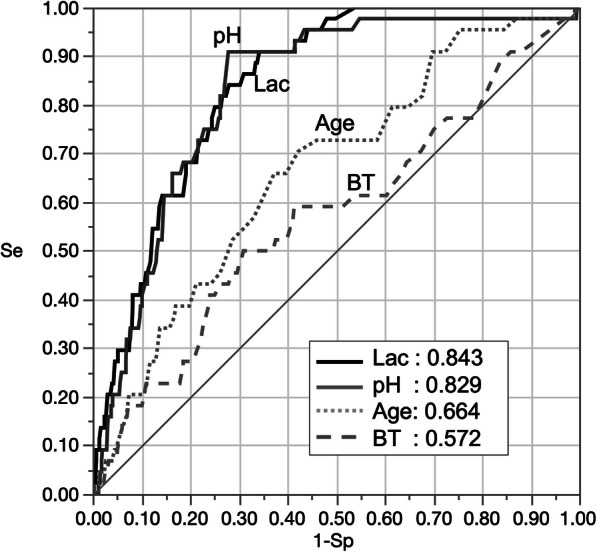
Receiver operating curve and Area under the curve for 1-month survival. Lac, serum lactate; BT, body temperature; Se, sensitivity; Sp, specificity

The AUC with 95% CI of the predictors for 1-month survival were as follows: Age 0.664 [0.579–0. 739], BT 0.573 [0.477–0.663], pH 0.829 [0.767–0.877], and lactate 0.843[0.793–0.882] (Fig. 2). The predictive ability in lactate and pH were described in Tables 3, and 4. On setting the cutoff points of 6.9 in pH, and 120 mg/dL (=13.3 mmol/L) in lactate, the predictors had a high sensitivity (lactate: 0.91, pH 0.91) and a low LR- (lactate: 0.14, pH 0.13), which are suitable to rule-out 1-month survival.

**Table 3:** The predictive accuracy of lactate level for 1-month survival

Cutoff (mg/dL)	Se	Sp	TP	TN	FP	FN	LR+	LR-	PPV	NPV
40	0.30	0.93	13	612	45	31	4.3	0.76	0.22	0.95
60	0.45	0.89	20	582	75	24	4.0	0.62	0.21	0.96
80	0.61	0.82	27	540	117	17	3.4	0.47	0.19	0.97
100	0.80	0.74	35	484	173	9	3.0	0.28	0.17	0.98
120	0.91	0.64	40	419	238	4	2.5	0.14	0.14	0.99
140	0.98	0.51	43	334	323	1	2.0	0.04	0.12	1.00

*TP* True-positive, *TN* True-negative, *FP* False-positive, *FN* False-negative, *Se* Sensitivity, *Sp* Specificity, *LR+* Positive likelihood ratio, *LR* Negative likelihood ratio, *PPV* Positive predictive value, *NPV* Negative predictive value

**Table 4:** The predictive accuracy of pH for 1-month survival

Cutoff	Se	Sp	TP	TN	FP	FN	LR+	LR-	PPV	NPV
7.3	0.14	0.97	6	640	20	38	4.5	0.89	0.23	0.94
7.2	0.20	0.95	9	625	35	35	3.9	0.84	0.20	0.95
7.1	0.43	0.89	19	589	71	25	4.0	0.64	0.21	0.96
7.0	0.68	0.81	30	537	123	14	3.7	0.39	0.20	0.97
6.9	0.91	0.70	40	464	196	4	3.1	0.13	0.17	0.99
6.8	0.95	0.53	42	349	311	2	2.0	0.09	0.12	0.99
6.7	0.98	0.35	43	229	431	1	1.5	0.07	0.09	1.00

*TP* True-positive, *TN* True-negative, *FP* False-positive, *FN* False-negative, *Se* Sensitivity, *Sp* Specificity, *LR+* Positive likelihood ratio, *LR* Negative likelihood ratio, *PPV* Positive predictive value, *NPV* Negative predictive value

### Additional analysis for the predictive accuracy of potassium

The potassium values were not reported in approximately half of the population included in the primary analysis. Thus, we did not calculate the predictive accuracy of potassium in the primary analysis. In additional analysis, we also excluded the patients who had missing data on potassium values from the cohort for primary analysis, hence, 458 patients from 66 hospitals (tertiary centers 56, non-tertiary centers 10 hospitals) were included in the additional analysis for the predictive accuracy of potassium. The 1-month survival was 5.5% (25/458). Details of this analysis are shown in the supplementary materials. The patient characteristics were found to be similar to those in the primary analysis. The AUC values [95% CI] of the potassium were as follows: 0.840 [0.757–0.898]. On setting the cutoff points of 7.0 (mmol/L) in serum potassium, it had the high sensitivity (0.96) and a low LR- (0.09), which are suitable to rule-out one-month survival.

## Discussion

### Key observation

From a large-scale hospital-based registry in Japan, we demonstrated the predictive accuracy of lactate, pH, and potassium for 1-month survival in OHCA patients with moderate-to-severe hypothermia who were transported to emergency departments. These predictors may be helpful to consider the resuscitation strategy.

### Previous literature and strengths

Our study has several advantages compared to previous studies. First, this is the first study to include a substantially large sample size to assess the predictive accuracy of pH, lactate, and potassium on 1-month survival outcome among OHCA patients with hypothermia who were transported to emergency departments. Most of the previous literature involves case-series or cohort studies with a small sample size; thus, the accuracy of these predictors could not be accurately assessed earlier [7–10]. Our results are more valid and robust than the prediction models that were previously reported. Recently, the HOPE and ICE scores were developed using a logistic regression model to predict the outcome among OHCA patients with hypothermia [25, 26]. However, the dataset of these studies were derived from a systematic review of published case reports, case series, and observational studies that had small sample sizes. We believe that their results were limited by risk of selection and publication bias. Furthermore, the researchers used a logistic regression model without a validation dataset. Therefore, the predictive accuracy may be optimistic and biased. Second, our study comprised a nationwide multicenter cohort that involved 87 institutions in Japan, at the forefront of super-aging societies, and it included many elderly OHCA patients. Thus, our results could be more generalizable to urban settings or aging societies than the previous studies, which included mainly young populations associated with outdoor activities. Based on these strengths, our study is more beneficial to the research question of the outcome than the abovementioned previous studies.

### Interpretation

We suggested some potential rationale for our results. In general, anaerobic glycolysis due to inadequate oxygen delivery causes lactate and metabolic acidosis [27, 28]. Furthermore, insufficient discharge of carbon dioxide due to low venous return and inappropriate ventilation provokes respiratory acidosis [29], which leads to the movement of potassium from the intracellular to the extracellular compartment [30]; thus, a longer duration of resuscitation is indicated by higher potassium levels in cardiac arrest patients [31]. Therefore, low pH value and higher lactate and potassium are indicative of hypoperfusion in vital organs, and longer duration of the resuscitation. Moreover, elevated potassium levels are associated with cell lysis after cell death, and it may be indicative of the futility of resuscitation attempts [10, 30, 31] Thus, it is reasonable that pH values, lactate, and potassium levels can accurately predict their survival.

### Clinical implication

We suggest considering pH, lactate, and potassium levels when planning resuscitation strategies, such as the termination of resuscitation or implementation of ECLS. In particular, OHCA patients with hypothermia who have a pH value lower than 6.9, lactate higher than 120 mg/dL, or potassium higher than 7 mmol/L have a high sensitivity for mortality, and termination of resuscitation may be acceptable in these patients. The results of blood tests are objective, reproducible, and available immediately on hospital arrival. Therefore, our results can be easily applied to clinical settings.

It should be noted that there are a few reports of hypothermic cardiac arrest cases in young patients with good recovery, despite severe acidemia or hyperkalemia [5, 8, 32]. These cases might be extremely rare; however, clinicians should not arrive at conclusions too quickly based only on the results of these predictors. We recommend that clinicians comprehensively evaluate the decision for advanced resuscitation or termination based on all the available information.

### Limitations

Our study has several limitations. First, there might be a bias in the measurement of important variables. This is because the timing of the blood test or procedure for the BT measurement was not strictly defined in the research protocol. Furthermore, during resuscitation, the PaCO_2_ value was changeable, subject to the administration of bicarbonate, ventilation, and venous return, and this may have influenced the pH and serum potassium levels. Second, some cases were excluded due to missing BT or blood test data. This might have potentially led to a bias in patient selection. Third, although our study was the largest cohort of cardiac arrest with hypothermia, the number of favorable neurological outcome was limited. Thus, we set the 1-month survival as the primary outcome; however, clinicians ideally hope to predict the neurological outcome. In terms of that, the clinical importance was slightly limited. Fourth, the most important limitation of this study was that the results of blood tests might have influenced the decision-making of each physician in charge of the patient with regard to the resuscitation strategies, acting similarly as a self-fulfilling prophecy. In other words, the result might only indicate that the lactate level was so high that the clinicians decided to terminate the resuscitation. Therefore, these limitations should be considered while interpreting our findings.. Therefore, further studies are warranted to assess the validity of our results.

## Conclusion

We indicated the predictive accuracy of pH, lactate, and potassium for 1-month survival among adult OHCA patients with moderate-to-severe hypothermia. In particular, pH 6.9, lactate 120 mg/dL (13.3 mmol/L), and potassium 7.0 mmol/L can be useful cutoff points to rule out 1-month survival with high sensitivity. Therefore, these factors might be considered as potential predictive biomarkers to consider the resuscitation strategy.

## Supplementary information

**Additional file 1.**

## Data Availability

Not applicable.

## Abbreviations

OHCA: Out-of-hospital cardiac arrest
JAAM: Japanese association of acute medicine
BT: Body temperature
V-A ECMO: Veno-arterial extracorporeal membrane oxygenation
ROSC: Return of spontaneous circulation
PEA: Pulseless electrical activity
VT: Ventricular tachycardia
ECMO: Extracorporeal membrane oxygenation
ICU: Intensive care unit
