# Supplementary material for: Predictive accuracy of biomarkers for survival among cardiac arrest patients with hypothermia: a prospective observational cohort study in Japan
Source: Scand J Trauma Resusc Emerg Med. 2020 Aug 5;28:75. doi: 10.1186/s13049-020-00765-2 (PMC7404926; doi:10.1186/s13049-020-00765-2)
Supplement: Supplementary file 1 — Additional file 1. [file 13049_2020_765_MOESM1_ESM.docx]

**Supplementary file**

**Definition of the area**

Each area is defined as below based on the definition of the Japan meteorological agency.[1]

Northern area: Hokkaido, and Tohoku region

Eastern area: Kanto, Hokuriku, and Tokai region

Western area: Kinki, Shikoku, Chugoku, and north side of Kyusyu

Southern area: Okinawa

**S-Fig 1. Definition of the area**


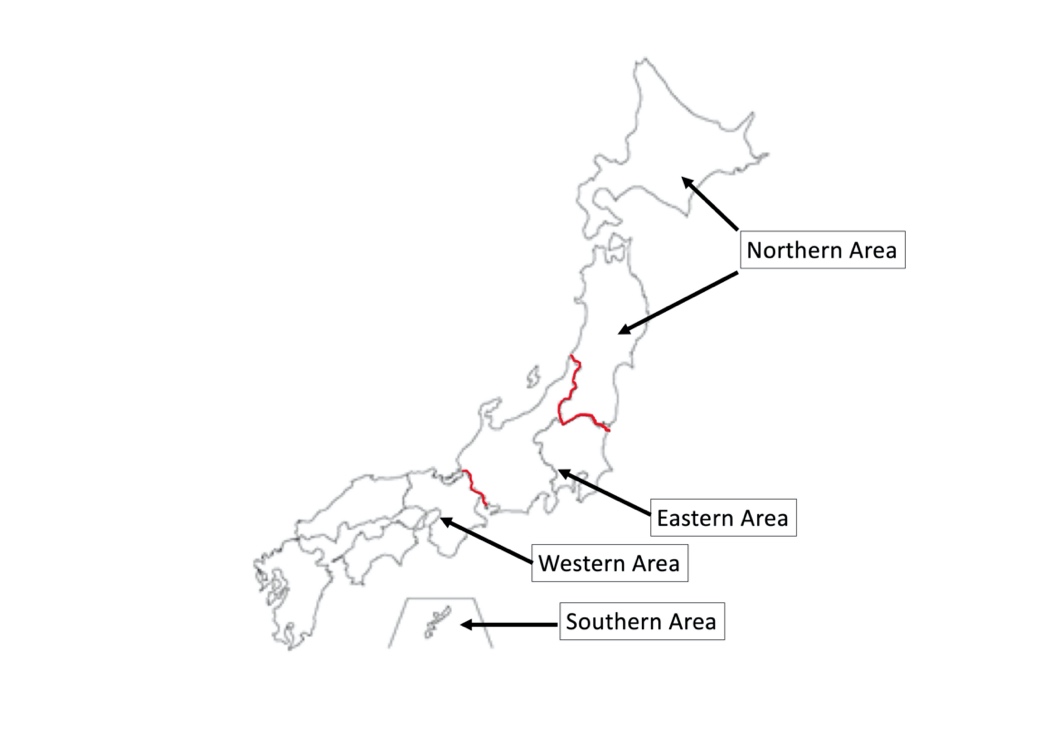


**S-Table 1. The mean temperature of the largest city in each region in 2017.** [1]

|  | Jan | Feb | Mar | April | May | June | July | Aug | Sep | Oct | Nov | Dec |
| --- | --- | --- | --- | --- | --- | --- | --- | --- | --- | --- | --- | --- |
| Northern | -3.9 | -2.0 | 1.4 | 7.7 | 14.4 | 16.0 | 22.9 | 21.7 | 17.7 | 11.3 | 4.3 | -2.0 |
| Eastern | 5.8 | 6.9 | 8.5 | 14.7 | 20.0 | 22.0 | 27.3 | 26.4 | 22.8 | 16.8 | 11.9 | 6.6 |
| Western | 6.2 | 6.3 | 9.2 | 15.7 | 21.1 | 22.7 | 28.8 | 29.2 | 24.4 | 18.4 | 12.6 | 7.0 |
| Southern | 18.4 | 17.1 | 18.3 | 21.6 | 24.2 | 26.6 | 29.9 | 30.4 | 28.9 | 27.0 | 22.8 | 18.0 |

Northern area: Sapporo, Eastern area: Tokyo, Western area: Osaka, Southern area: Naha

Temperature (℃)

**Additional analysis**

**Results**

Among the 34,754 patients in the JAAM-OHCA database, 458 patients from 54 hospitals (tertiary center 45, non-tertiary center 9) were included in the additional analysis (S-Fig 2). The characteristics of the patients and in-hospital data were described in S-Table 2 and 3. In summary, the median [IQR] of the age are 75 [64-84] years, and almost half of the cases happened in winter (222/458, 48.5%), and most of the cases occurred except for summer (405/458, 88.4% in spring, autumn, and winter). The median [IQR] of core BT were 29.7 [26.0-31.2]. The one-month survival was 5.5% (25/458).

**Predictive accuracy**

The AUC values [95% CI] of the potassium were 0.840 [0.757-0.898]. The other AUC values [95%CI] were as follows: BT 0.650 [0.53-0.754], Age 0.655 [0.537-0.757], Lactate 0.854 [0.790-0.900], and pH 0.822 [0.722-0.892], (S-Fig3). The predictive ability in potassium were described in S-Tables 4. On setting the cutoff points of 7.0 (mmol/L) in serum potassium, it had the high sensitivity 0.96 [95%CI: 0.87-1.00] and a low LR- 0.09, which are suitable to rule-out one-month survival.

**S-Fig 2. The study flowchart in additional analysis**

**
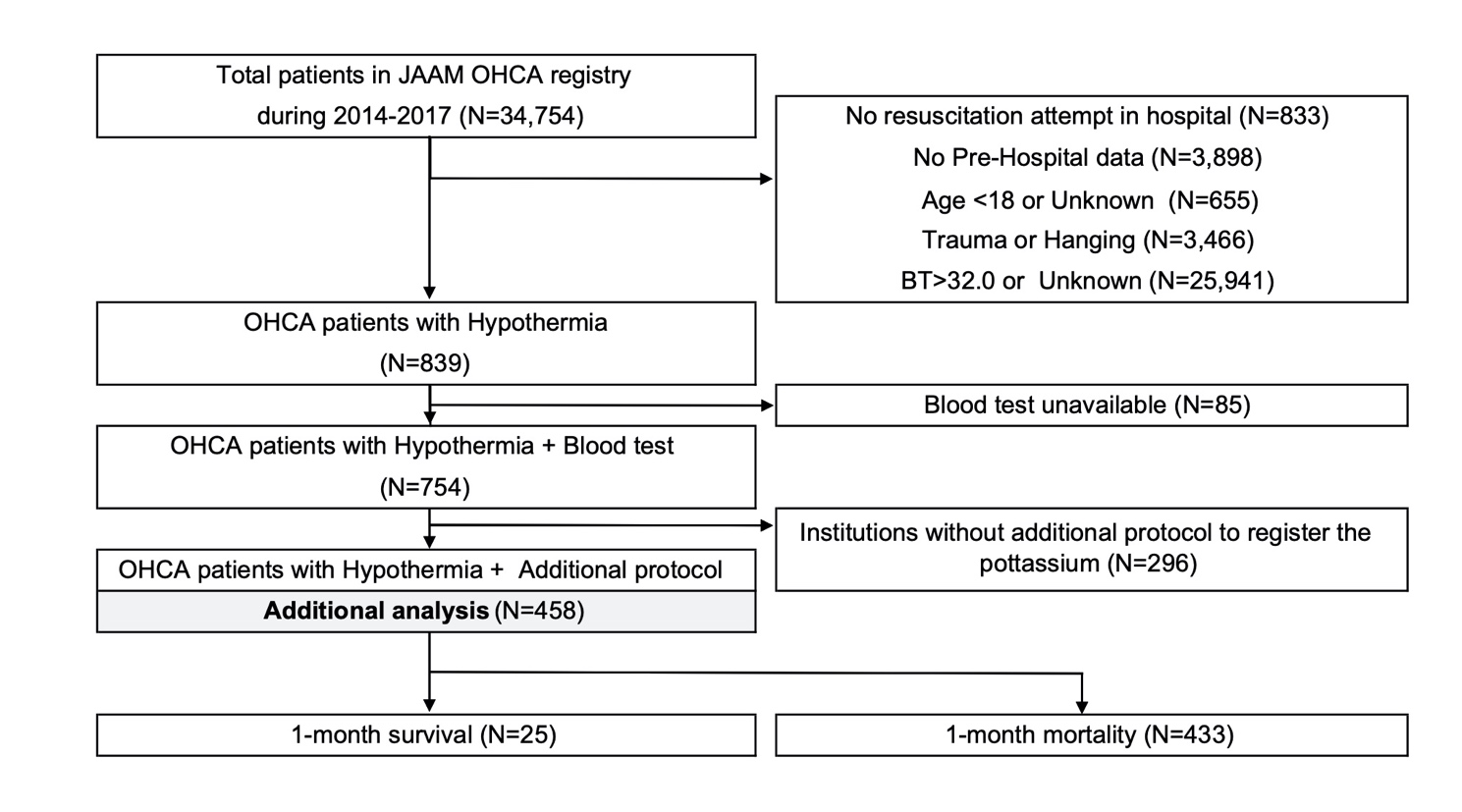
**

**S-Table 2. The characteristics of the patients in the additional analysis**

| Variables | | Total |
| --- | --- | --- |
|  |  | (N=458) |
| **Baseline characteristics** | |  |
| Sex (Men) | | 265 (57.9%) |
| Age (year) | | 75 [64-84] |
|  | 16-64 | 122 (26.6%) |
|  | 65-74 | 103 (22.5%) |
|  | ≥ 75 | 233 (50.9%) |
| **Pre-hospital information** | |  |
| Bystander witness | | 126 (27.5%) |
| Bystander CPR | | 161 (35.2%) |
| Shockable on initial rhythm | | 39 (8.5%) |
| Advanced airway | | 218 (47.6%) |
| **In-hospital information** | |  |
| Body temperature | | 29.7 [26-31.2] |
| Measurement site | |  |
|  | Rectal | 93 (20.3%) |
|  | Bladder | 84 (18.3%) |
|  | Tympanic | 48 (10.5%) |
|  | Other/Unknown | 233 (50.9%) |
| Cardiac rhythm on hospital arrival | |  |
|  | ROSC | 17 (3.7%) |
|  | Shockable | 40 (8.7%) |
|  | PEA | 80 (17.5%) |
|  | Asystole | 321 (70.1%) |
| ECMO implementation | | 41 (9%) |
|  | Before ROSC | 34 (82.9%) |
| ROSC after hospital arrival | | 97 (21.2%) |
| Time course (min) | |  |
|  | E-call to Hospital arrival | 34 [28-42] |
|  | E-call to Blood test | 40 [34-52] |
|  | E-call to ECMO | 70 [52.3-88] |
|  | E-call to ROSC after arrival | 57 [40-96] |
| Blood test on hospital arrival | |  |
|  | pH | 6.8 [6.6-7] |
|  | (Missing) | 35 (7.6%) |
|  | Lactate (mg/dl) | 131.4 [79.2-180] |
|  | (Missing) | 39 (8.5%) |
|  | Potassium (mmol/l) | 6.6 [4.9-9.6] |
|  | (Missing) | 87 (19%) |
| Outcome | |  |
|  | Admission to ICU or ward | 101 (22.1%) |
|  | Death in ER | 357 (77.9%) |
|  | 1-month Survival | 25 (5.5%) |
|  | 1-month CPC1,2 | 15 (3.3%) |

Continuous variables are described as median [Interquartile range (IQR)]. Categorical variables are described as number (%). Shockable: ventricular fibrillation and pulseless ventricular tachycardia, CPR: Cardiopulmonary resuscitation, E-call: Emergency call for ambulance, ROSC: Return of spontaneous circulation, PEA: Pulseless electrical activity, ECMO: Extracorporeal membrane oxygenation, ER: Emergency room, CPC: Cerebral performance category[2]

**S-Table 3. The other characteristics of the patients in the additional analysis**

| **Variables** | | Total |
| --- | --- | --- |
|  |  | (N=458) |
| **Hospital Information** | |  |
| **Hospital** | |  |
|  | Tertiary center (45 hospitals) | 432 (94.3%) |
|  | Non-tertiary center (9 hospitals) | 26 (5.7%) |
|  | The number of beds | 707 [388-938] |
| **ECMO availability** | |  |
|  | Always | 377 (82.3%) |
|  | Partial | 61 (13.3%) |
|  | Unavailable | 20 (4.4%) |
| **Geographical information and season** | |  |
| **Area** | |  |
|  | Northern area | 139 (30.4%) |
|  | Eastern area | 136 (29.7%) |
|  | Western area | 183 (40%) |
|  | Southern area | 0 (0%) |
| **Season** | |  |
|  | Spring | 85 (18.6%) |
|  | Summer | 53 (11.6%) |
|  | Autumn | 98 (21.4%) |
|  | Winter | 222 (48.5%) |

Continuous variables are described as median [Interquartile range (IQR)]. Categorical variables are described as number (%).Shockable: ventricular fibrillation and pulseless ventricular tachycardia

E-call: Emergency call for ambulance, ROSC: Return of spontaneous circulation, ECMO: Extracorporeal membrane oxygenation, ER: Emergency room

**S-Figure 2. Receiver operating curve and Area under the curve for one-month survival.**


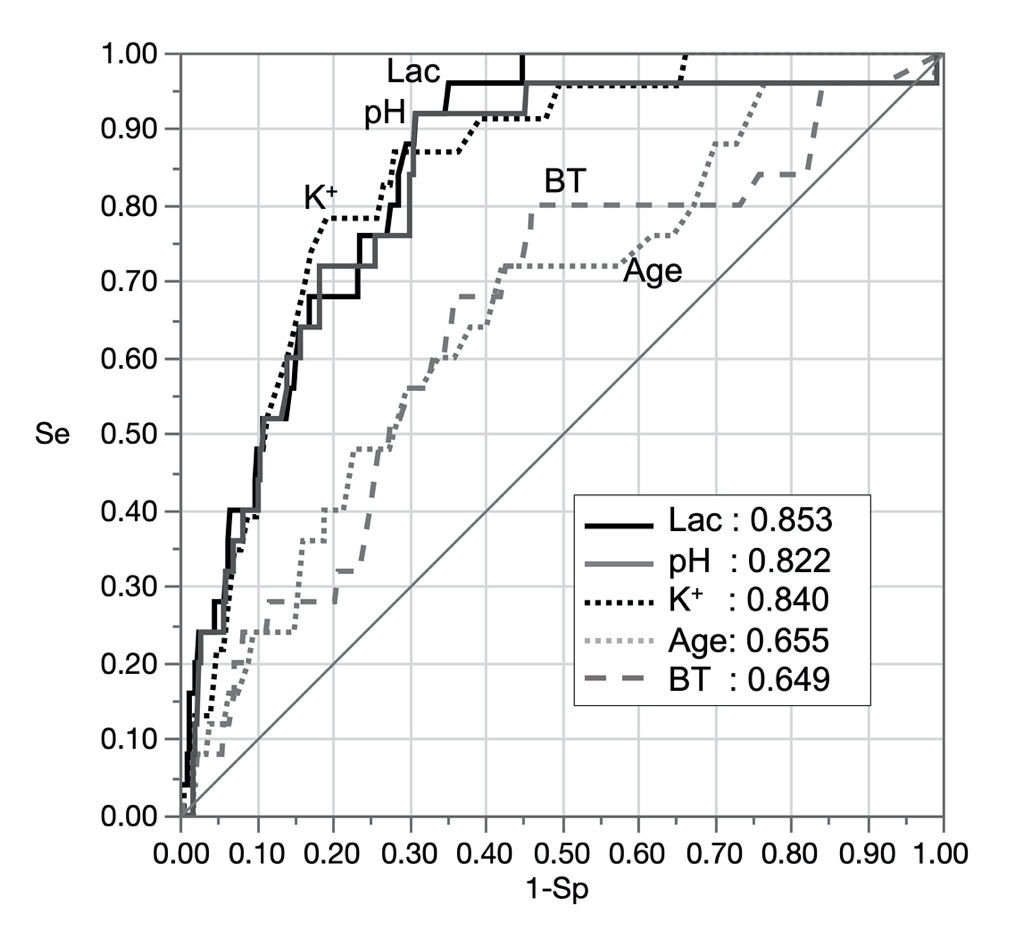


K^+^: Serum potassium, Lac = serum lactate, BT = body temperature, Se: Sensitivity, Sp: Specificity

**S-Table 4. The predictive accuracy of potassium for 1-month survival**

| **Cut-off (mmol/l)** | **Se** | **Sp** | **TP** | **TN** | **FP** | **FN** | **LR+** | **LR-** | **PPV** | **NPV** |
| --- | --- | --- | --- | --- | --- | --- | --- | --- | --- | --- |
| 4.0 | 0.48 | 0.90 | 11 | 312 | 36 | 12 | 4.6 | 0.58 | 0.23 | 0.96 |
| 5.0 | 0.78 | 0.77 | 18 | 267 | 81 | 5 | 3.4 | 0.28 | 0.18 | 0.98 |
| 6.0 | 0.87 | 0.64 | 20 | 221 | 127 | 3 | 2.4 | 0.21 | 0.14 | 0.99 |
| 7.0 | 0.96 | 0.49 | 22 | 172 | 176 | 1 | 1.9 | 0.09 | 0.11 | 0.99 |
| 8.0 | 0.96 | 0.39 | 22 | 136 | 212 | 1 | 1.6 | 0.11 | 0.09 | 0.99 |
| 9.0 | 1.00 | 0.31 | 23 | 109 | 239 | 0 | 1.5 | 0.00 | 0.09 | 1.00 |
| 10.0 | 1.00 | 0.21 | 23 | 72 | 276 | 0 | 1.3 | 0.00 | 0.08 | 1.00 |

TP: True-positive, TN: True-negative, FP: False-positive, FN: False-negative, Se: Sensitivity, Sp: Specificity, LR+: Positive likelihood ratio, LR-: Negative likelihood ratio, PPV: Positive predictive value, NPV: Negative Predictive value

**Reference**

1. **Japan Meteorological Agency** [<https://www.jma.go.jp/jma/indexe.html>]

2. Cummins RO, Chamberlain DA, Abramson NS, Allen M, Baskett PJ, Becker L, Bossaert L, Delooz HH, Dick WF, Eisenberg MS *et al*: **Recommended guidelines for uniform reporting of data from out-of-hospital cardiac arrest: the Utstein Style. A statement for health professionals from a task force of the American Heart Association, the European Resuscitation Council, the Heart and Stroke Foundation of Canada, and the Australian Resuscitation Council**. *Circulation* 1991, **84**(2):960-975.
